# Accelerated development in Johnsongrass seedlings (*Sorghum halepense*) suppresses the growth of native grasses through size-asymmetric competition

**DOI:** 10.1371/journal.pone.0176042

**Published:** 2017-05-03

**Authors:** Susanne Schwinning, Heather Meckel, Lara G. Reichmann, H. Wayne Polley, Philip A. Fay

**Affiliations:** 1 Department of Biology, Texas State University, San Marcos, Texas, United States of America; 2 Department of Integrative Biology, University of Texas at Austin, Austin, Texas, United States of America; 3 USDA-ARS Grassland, Soil and Water Research Laboratory, Temple, Texas, United States of America; Instituto Agricultura Sostenible, SPAIN

## Abstract

Invasive plant species often dominate native species in competition, augmenting other potential advantages such as release from natural enemies. Resource pre-emption may be a particularly important mechanism for establishing dominance over competitors of the same functional type. We hypothesized that competitive success of an exotic grass against native grasses is mediated by establishing an early size advantage. We tested this prediction among four perennial C_4_ warm-season grasses: the exotic weed Johnsongrass (*Sorghum halepense*), big bluestem (*Andropogon gerardii*), little bluestem (*Schizachyrium scoparius*) and switchgrass (*Panicum virgatum*). We predicted that a) the competitive effect of Johnsongrass on target species would be proportional to their initial biomass difference, b) competitive effect and response would be negatively correlated and c) soil fertility would have little effect on competitive relationships. In a greenhouse, plants of the four species were grown from seed either alone or with one Johnsongrass neighbor at two fertilizer levels and periodically harvested. The first two hypotheses were supported: The seedling biomass of single plants at first harvest (50 days after seeding) ranked the same way as the competitive effect of Johnsongrass on target species: Johnsongrass < big bluestem < little bluestem/switchgrass, while Johnsongrass responded more strongly to competition from Johnsongrass than from native species. At final harvest, native plants growing with Johnsongrass attained between 2–5% of their single-plant non-root biomass, while Johnsongrass growing with native species attained 89% of single-plant non-root biomass. Fertilization enhanced Johnsongrass’ competitive effects on native species, but added little to the already severe competitive suppression. Accelerated early growth of Johnsongrass seedlings relative to native seedlings appeared to enable subsequent resource pre-emption. Size-asymmetric competition and resource-pre-emption may be a critical mechanism by which exotic invasive species displace functionally similar native species and alter the functional dynamics of native communities.

## Introduction

Invasive alien species often establish in communities where they compete against native species of the same plant functional type. An example with worldwide relevance is the invasion of mid-latitude C_4_ grasslands by alien C_4_ grasses [1–5]. Although grassland species may be expected to persist in any climatically similar grassland worldwide, it is often not apparent what traits enable new arrivals to spread rapidly and replace functionally similar native species [6]. Though a difference in interactions with herbivores and pathogens may play a role [7], controlled experiments often show that invasive species are competitively dominant over native species [8, 9]. Two mechanisms may contribute to invasive success [10, 11]: an elevated level of competitive effect, by which invasive species reduce native species growth and fitness, and a reduced competitive response to native species, which would allow invasive species to thrive in the presence of native competitors.

In grassland communities, competitive effect and response are frequently uncoupled—low competitive response does not predict high competitive effect and vice versa [12]. However, competition between functionally similar species, with widely overlapping niches, may be constrained by a zero-sum game of resource capture, as the resources left unused by one species may be swept up by another [13]. Among such ‘functionally redundant’ species [14], competitive response and effect may be two sides of the same coin. Consequently, an invasive species with the ability to pre-empt jointly limiting resources would have a large competitive effect on native neighbors and by keeping natives resource-starved and small, also a small competitive response.

Size hierarchies readily establish in monocultures and species mixtures through the amplification of small size differences among seedlings [15, 16]. The key to the establishment of size hierarchies is asymmetric resource competition, which increases the per-biomass interception of a limiting resource—typically light—by taller individuals at the cost of light interception by shorter, shaded, neighbors [17]. Thus, to win an open site, an invasive species may only have to produce larger seedlings at the beginning of the growing season to get ahead of native species occupying the same niche. Accordingly, ‘size’ [12, 18, 19], early arrival or germination (‘priority’) [20, 21] or accelerated seedling development [22] are often found to be among the distinct characteristics of invasive species when they are compared with functionally equivalent native species. For example, a recent study showed that invasive and native annual plants, lacking differences in functional traits including assimilation rate and resource use efficiencies, did differ in the size-related traits including seed mass and stature [6]. This suggests that if an invasive species competing with functionally similar natives can establish an initial size advantage, and resource competition is asymmetric, its successful establishment and disproportional accumulation of biomass [13] would seem to be assured.

Johnsongrass (*Sorghum halepense*) is an invasive exotic grass and noxious weed with worldwide distribution. In North America, it invades agricultural fields [23–25] and natural grasslands [26], including those dominated by functionally similar warm season perennial C_4_ grasses. Early germination is not necessarily expected from Johnsongrass, since it requires higher temperature for germination (> 10°C; [26]) than many prairie species such as big bluestem (*Andropogon gerardii*) and switchgrass (*Panicum virgatum*), whose base temperature for germination is lower (< 10°C [27, 28]). However, we previously showed that when these three species and little bluestem (*Schiachyrium scoparium*) were germinated simultaneously at 20–25°C in the greenhouse, Johnsongrass attained a fourfold size advantage over the native species in just 17 days [29].

Here, we ask how this size disparity translates into competitive interactions between Johnsongrass and three native grasses. Growing plants from seed in deep containers in a greenhouse, we simulated seedling competition for open sites. We hypothesized that in these four C_4_ grass species with ostensibly shared functional strategies, size-asymmetric competition would dominate Johnsongrass’ competitive success, irrespective of soil nutrient regime. Although C_4_ species have a measure of trait diversity, including in nitrogen use efficiency reflecting their independent evolution [30], we hypothesized that a difference in nitrogen use is not the main cause of competitive dominance in Johnsongrass. Based on this, we predicted that 1) the competitive effect of Johnsongrass on target species would be proportional to their initial biomass difference; 2) Johnsongrass’ competitive response and effect are inversely correlated with each other and 3) nitrogen availability will not greatly alter the competitive dominance of Johnsongrass. During the experiment, we also imposed a mid-summer drought by withholding irrigation water to establish conditions closer to field conditions, where soil moisture typically fluctuate and might complicate relationships between plant size and growth.

## Materials and methods

### Experimental design

Seeds of Johnsongrass *(Sorghum halepense*), big bluestem (*Andropogon gerardii* var. Kaw), little bluestem (*Schizachyrium scoparius*) and switchgrass (*Panicum virgatum* var. ‘Alamo’), all warm-season, C_4_, perennial bunch grasses, were obtained from commercial sources (natives: Native American Seed, Junction, TX; and Johnsongrass: Turner Seed Co., Breckenridge, TX).

Plants were grown from seed in a climate-controlled greenhouse at Texas State University in San Marcos, Texas, from 7 June to 9 December 2011. Seeds were planted in long polyethylene containers (100 cm deep and 11.3 cm wide) to minimize vertical restrictions to root growth. Containers were filled with 10 liters of a 70:30 mixture of sand to local topsoil. The high sand ratio was chosen to facilitate root extraction. Nutrient content was supplemented with either 1 or 3 g slow release fertilizer (Osmocote, 14-14-14 NPK, The Scotts Company LLC, Marysville, OH) applied to the top 10 cm of the soil. This fertilizer treatment was selected to establish the nitrogen levels within the range of local topsoil and avoid limitation by other nutrients [31]. Containers were lined with reflective bubble wrap to control temperature and prevent algal growth.

The experiment consisted of a complete factorial, randomized block design with four factors: species (at four levels: big bluestem, little bluestem, switchgrass and Johnsongrass), Competition (at two levels: a single seedling to a pot or one seedling paired with a Johnsongrass seedling), fertilizer (at two levels: one or three grams Osmocote per pot) and harvest date (at four levels: 49, 71, 100 and 183 days from seeding). Thus, all four species were grown either alone or in combination with Johnsongrass at two fertilizer levels. We refer to the two plants in the paired-plant treatment as the ‘focal plant’ and the ‘Johnsongrass neighbor’. Four blocks were arranged in rows at increasing distance from the greenhouse’s evaporative cooler and each block contained at least four replicates per treatment combination. At each harvest, one complete suite of samples was randomly selected from each block. Additional replicates for single plants allowed us to conduct one additional harvest for single plants.

### Growing conditions

Before seeding, the soil columns were saturated with water top to bottom. On 8 June (day 0), approximately ten seeds per species were dropped into containers and lightly covered with soil. Emergence was checked daily and three seedlings per subject were maintained until the first true leaf emerged, after which they were thinned to one plant per subject. Seedlings from late-germinating seeds were removed daily. Containers were watered daily from a hand-held hose to keep the top 10 cm of soil saturated. The infiltration depth could be seen through the clear plastic.

Beginning 50 days after seeding, watering was ceased for 50 days, from July 28 –September 15 to simulate a mid-summer drought, a common occurrence in Central Texas. Afterwards, the daily watering schedule was resumed until the experiment ended after 184 days, on December 9. Throughout the experiment, the greenhouse temperature and humidity settings were adjusted to maintain warm conditions (30–35°C), representative of spring to early fall conditions in central Texas.

### Leaf and plant traits

Leaf and whole plant traits related to water stress and nutrient uptake and allocation were measured to evaluate potential underlying mechanisms for size-related competition. The predawn water potential of plants (Ψ_pre_) reflect soil water potential in the primary root zone [32], thus can provide a measure of soil water availability. Leaf conductance values are a measure of physiological response to limiting soil water, as plants reduce stomatal aperture to avoid wilting. We measured both variables to examine plant responses to the mid-summer drought 21 and 22 days into the drought (before the second harvest), 49 and 50 days into the drought (before the third harvest) and 35 and 36 days after the drought ended. After 50 days of drought, many plants did not have live leaves to measure and this reduced the sample number in some cases to 1 or 0.

We determined Ψ_pre_ using a Scholander pressure chamber on excised leaf blades (PMS 1000, PMS Instrument Co.,Albany, OR). Afterwards, from 10 am to approximately 3 pm, we took leaf gas exchange measurements characteristics, including leaf conductance and photosynthesis, using a LI-6400 fitted with a 6 cm^2^ cuvette and a blue-red LED light source (LiCor Biosciences, Lincoln, NE). Cuvette conditions were set near ambient greenhouse conditions (1000 μmol m^-2^ s^-1^ PAR, 400 μmol mol^-1^ external CO_2_ concentration, 35°C temperature).

### Harvest procedures

We harvested single and paired plants on 25–28 July (days 48–50), 17–19 August (days 70–72), 14–16 September (days 98–100) and 7–9 December (days 182–184). An additional harvest of single plants was conducted on 21–23 October (days 135–137).

At harvest, plant biomass was separated into rhizome, root, live stem, green leaf, senesced leaf and stem, and reproductive structures. Roots were separated from soil in two stages. In the first pass, the coarse root system was lifted out of the soil and washed in a sieve under running water. In the second pass, finer roots were sifted out of the soil by combing through the soil by hand. This was repeated until very little new root biomass was found. To standardize the capture efficiency between samples, root sifting ceased when 1 min of additional search yielded no additional root biomass. All roots captured from the two passes were combined and washed under running water. No attempt was made to separate the roots in the paired plant treatment.

The green leaf material of small plants was scanned and images analyzed with WinFOLIA (Regent Instruments, Inc., Quebec, Canada) to determine leaf area. For larger plants, the leaf area of a representative subsample was scanned and total leaf area was extrapolated from total green leaf biomass.

All biomass fractions were oven-dried at 70°C to a constant weight. A drying oven malfunction incinerated most green leaf area samples from the second harvest. We gap-filled missing data by estimating leaf weights from leaf areas using linear regression equations obtained from July data (big bluestem: R^2^ = 0.9704; Johnsongrass: R^2^ = 0.7687; little bluestem: R^2^ = 0.9824; switchgrass: R^2^ = 0.9862).

### Nitrogen content and concentration

Tissue nitrogen concentrations were determined for all plant materials collected during the first harvest. All biomass fractions were ground to a fine powder and subsamples of approximately 7 mg were weighed into 4 x 6 mm tin capsules (Costech Analytical Technologies, Inc., Valencia, CA) and assayed for carbon and nitrogen content in an elemental analyzer (Flash 2000, Thermo Scientific, Waltham, MA, USA) at the USDA/ARS lab in Temple, TX. Tissue N densities were calculated by dividing the N mass by the dry mass of the sample. Tissue specific nitrogen content was determined by multiplying each biomass fraction with the respective N density. Total plant nitrogen content was determined by summing the nitrogen content over all tissues.

### Data analysis

To determine species differences in growth in the absence of competition, we conducted an ANOVA of total biomass with harvest date, species and fertilizer as fixed factors. We calculated relative growth rates (RGR) as the increase in the average log biomass of species*fertilizer groups between two consecutive harvest dates, divided by the number of days between harvests. Since RGRs could not be determined for individual samples, we estimated the variance in RGR values by averaging the sample variances of the two log biomass sets (assuming zero covariance) and contrasted means using a two sample t-tests.

To quantify the competitive relationships between focal plants and Johnsongrass neighbors, we calculated competitive effects and responses as the log ratios of plants grown alone versus paired with Johnsongrass [33]. Because root biomass could not be separated in the paired plant treatment, we only included non-root biomass in these calculations. The competitive effect of Johnsongrass on a focal plant was calculated as

$$\text{log }CE_{X}=\text{log}\left(\bar{B_{X}}\right)-\text{log}(B_{X,J})$$ (1)

where *X* is the species index for the focal plant and *J* stands for the Johnsongrass neighbor. $\bar{B_{X}}$ is the average biomass value for single plants of species *X* and *B*_*X*,*J*_ is the biomass of a focal pant of species *X* growing with Johnsongrass at the respective fertilizer level and harvest date.

The competitive response of Johnsongrass to species *X* was calculated as

$$\text{log}CR_{X}=\text{log}(\bar{B_{J}})-\text{log}\left(B_{J,X}\right)$$ (2)

where the first term is the log of the average biomass of single Johnsongrass plants and *B*_*J*,*X*_ is the biomass of a Johnsongrass neighbor growing with a focal plant of species X at the respective fertilizer level and harvest date. Both indices were analyzed in a complete factorial ANOVA with species, fertilizer and harvest date as factors. However, Johnsongrass as focal plant was omitted from this analysis to focus on interspecific competition. Block effects were omitted since their effects on biomass harvest date was found to be insignificant. Regressions between log biomass and *log CE* and between *log CE* and *log CR* were conducted on species means.

To examine how nitrogen content and concentration were influenced by competition and fertilizer, we conducted ANOVAs on the total nitrogen content and the nitrogen concentration of all non-root tissue at first harvest. To simplify interpretation, this analysis was conducted by species. To examine how water availability may have mediated competitive interactions, we also conducted ANOVAs by species on Ψ_pre_ with harvest date, fertilizer and competition as factors. All statistical analyses were conducted with SPSS 20 IBM Corp., Armonk, NY).

## Results

### Species differences in size and growth for plants growing alone

**Fig 1:**
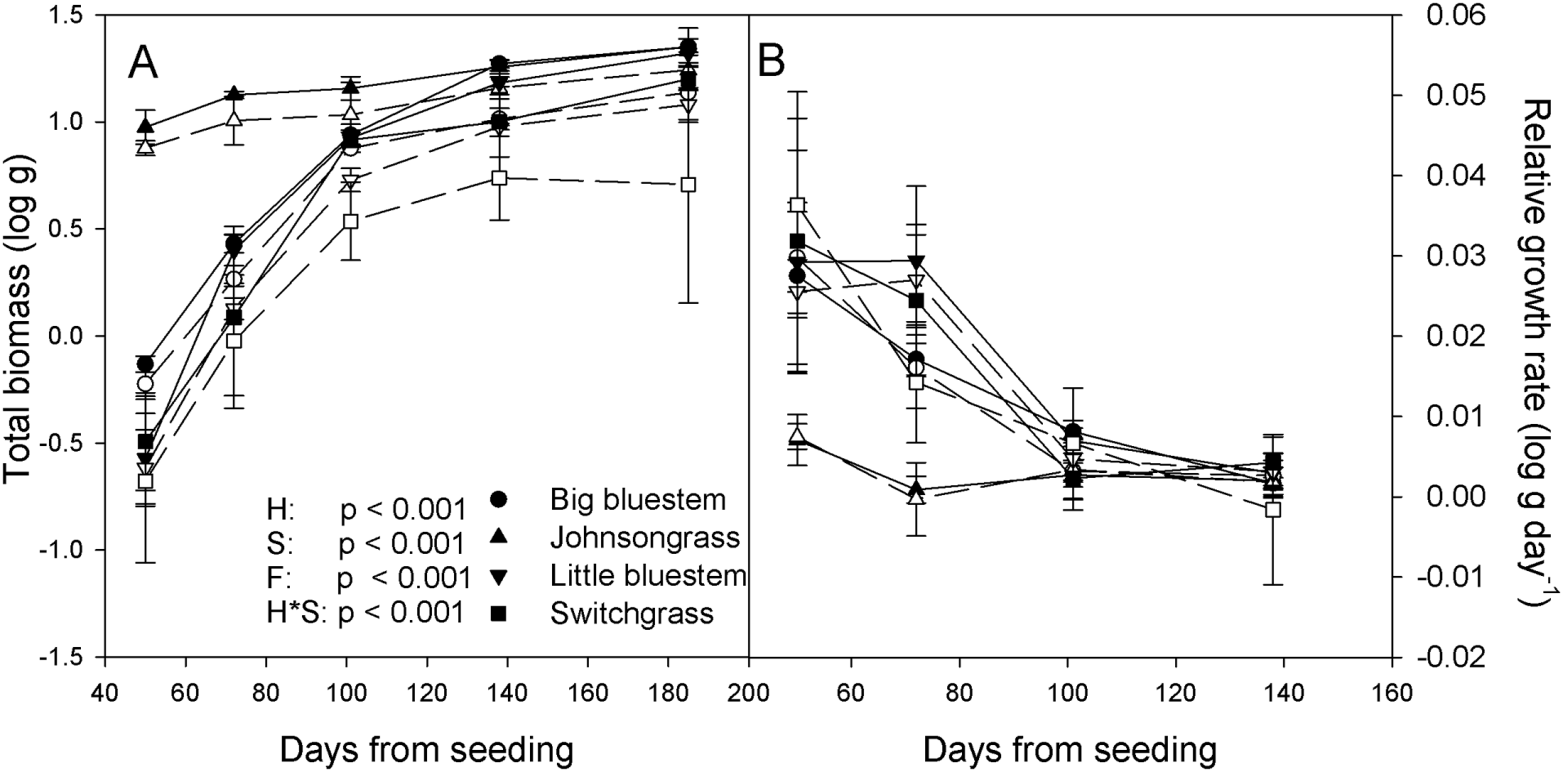
Growth dynamics of the four study species grown as single plants. A: Log of total biomass over five harvest dates. Shown in the panel are the p-values of all statistically significant effects, including harvest date (H), species (S), fertilizer (F) and a harvest date x species interaction (H*S). B: Relative growth rates for the interval between two consecutive harvests, standardized to a daily relative growth rate. Solid symbols for the higher fertilizer level, open symbols for the lower level. Error bars reflect one standard error of the mean.

There were size differences between the four grasses, maximal at the first harvest and declining thereafter (Fig 1A). Across all species and dates, fertilizer increased total biomass by 43% with no significant species x fertilizer interactions (p = 0.513). At first harvest, 50 days from seeding, Johnsongrass had between 13 and 30 times more biomass than the native species, significantly different from all native species (Tukey HSD p < 0.001 or p = 0.005 for the Johnsongrass—big bluestem contrast). Among the native species, biomass ranked big bluestem > switchgrass > little bluestem at first harvest and the size difference between the two bluestems was marginally significant (Tukey HSD p = 0.053). At final harvest, these size differences were reduced (species x harvest date p < 0.001), with Johnsongrass exceeding the non-root biomass of the native species only by a factor 1.4 on average and species differences were not significant (Tukey HSD p < 0.134).

Johnsongrass and the three native species clearly developed at different rates (Fig 1B). For example, on day 50, Johnsongrass, having already attained 42% of its final biomass, grew more slowly than the native species (two sample t-test p < 0.001). The native species reached between 40 and 46% of final biomass 50 days later, at the third harvest. By then, their relative growth rate was as low as that of Johnsongrass at first harvest (two-sample t-test p = 0.352).

#### Competitive effects and responses

**Fig 2:**
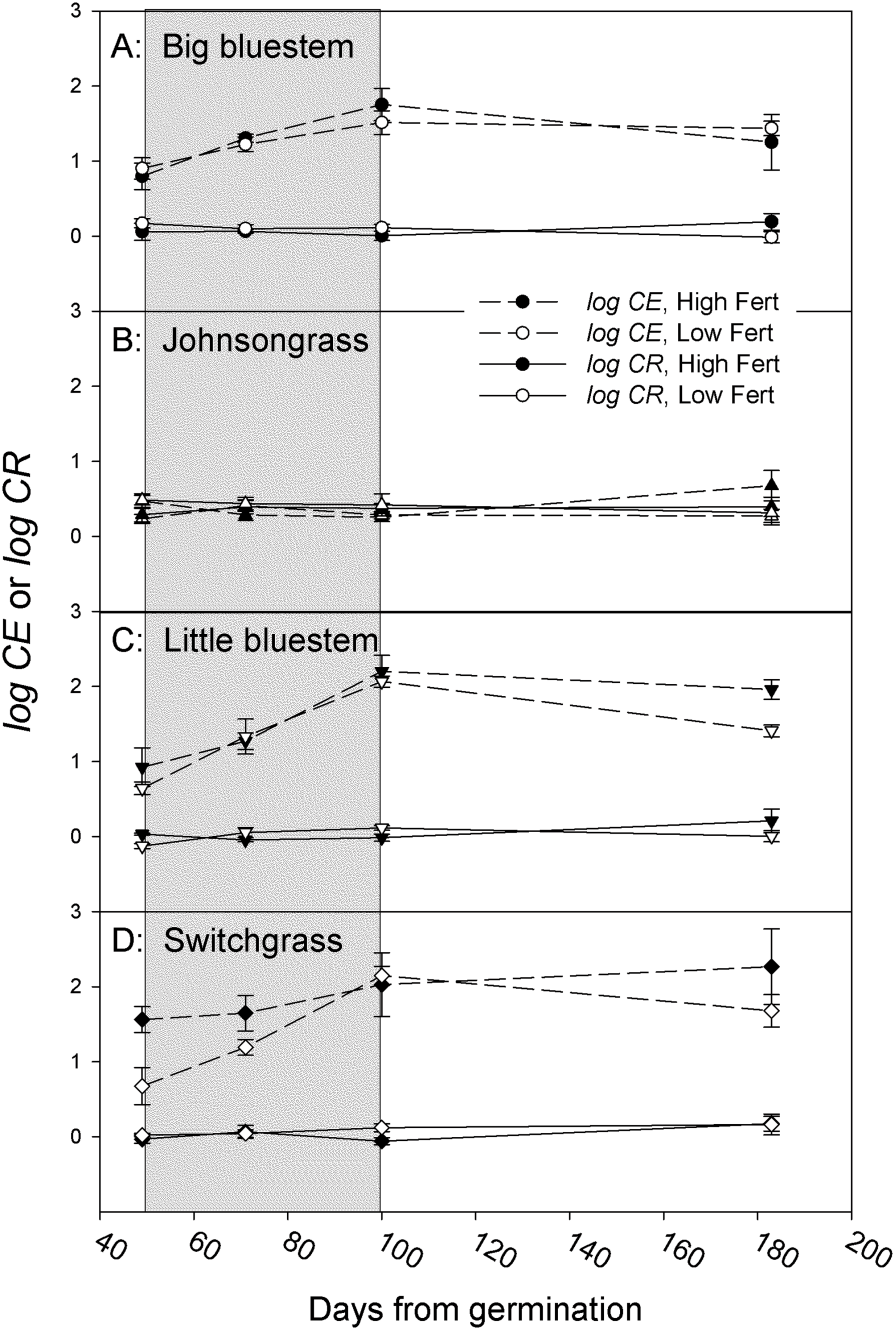
Competitive effects (*log CE*, dashed lines) and responses (*log CR*, solid lines) of Johnsongrass. Positive values indicate reduced non-root biomass relative to single plants. Solid symbols for high fertilizer, open symbols for low fertilizer. The shaded area indicates the period over which water was withheld. Error bars reflect one standard error of the mean.

The competitive effects of Johnsongrass on native species were already well established at first harvest (Fig 2, broken lines, Table 1A). At that time, big bluestem attained 14% of its single plant biomass, little bluestem 16% and switchgrass 9%. The competitive effect of Johnsongrass on the native species subsequently increased during the drought period (harvest date p < 0.001, Fig 2A, 2C and 2D) and became maximal at the end of the drought period. At final harvest, the non-root biomass relative to single plants was 5% for big bluestem and 2% for both little bluestem and switchgrass. Across harvest date and fertilizer levels, differences between the native species were significant (species p = 0.003; Table 1A).

**Table 1:** Competitive effect and response of Johnsongrass on focal plants examined by ANOVA. Johnsongrass as focal plant was omitted from this analysis to focus on interspecific competition. Significant effects are highlighted in bold lettering.

Source	df	MS	p
**A: *log CE* (Competitive Effect)**			
**Intercept**	**1**	**195.405**	**< 0.001**
**Species**	**2**	**1.068**	**0.003**
**Fertilizer**	1	**1.202**	**0.011**
**Harvest date**	**3**	**4.487**	**< 0.001**
Species x Fertilizer	2	0.377	0.124
Species x Harvest date	6	0.182	0.721
Fertilizer x Harvest date	3	0.094	0.660
Species x Fertilizer x Harvest date	6	0.223	0.232
Error	69	0.176	
Total	93		
**B: *log CR* (Competitive Response)**			
**Intercept**	**1**	**0.327**	**< 0.001**
Species	2	0.025	0.327
Fertilizer	1	0.002	0.781
Harvest date	3	0.043	0.131
Species x Fertilizer	2	0.013	0.558
Species x Harvest date	6	0.018	0.549
**Fertilizer x Harvest date**	**3**	**0.080**	**0.018**
Species x Fertilizer x Harvest date	6	0.019	0.526
Error	69	0.022	
Total	93		

We predicted that nitrogen availability would not greatly influence the competitive relationships between species. We found that, over the growing season and across species, the higher fertilizer level significantly increased the competitive effect (fertilizer p = 0.013; Table 1A). Specifically, the average effect of the higher fertilizer level was to reduce the biomass of native plants competing with Johnsongrass from 4.3% to 2.8% of single plant biomass.

The competitive response of Johnsongrass to the native species was small and did not change significantly over time, with species or consistently with fertilizer level (Fig 2, solid lines; Table 1B). A significant fertilizer x harvest date interaction (p = 0.018, Table 1B) could be attributed to the third harvest, when Johnsongrass competing with native species at the lower fertilizer level attained 76% of its single plant biomass and 105% at the higher fertilizer level. This effect may have been spurious and had dissipated at the final harvest when the average non-root biomass of Johnsongrass competing with native species was 89% that of single plants, irrespective of fertilizer level.

**Fig 3:**
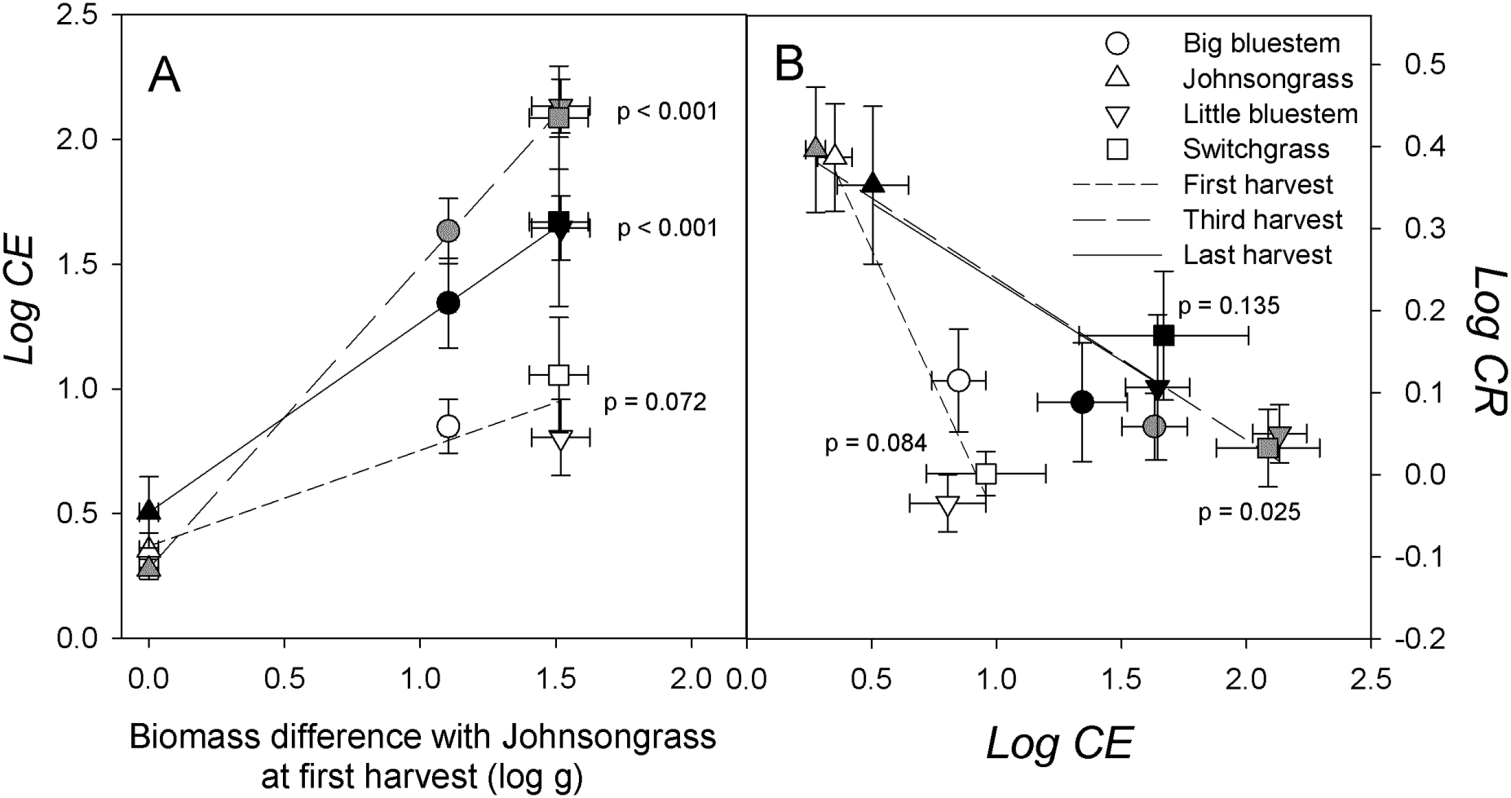
**A: Competitive effect (*log CE*) as a function of the log biomass difference between single plants and the average of Johnsongrass at the first harvest. B: Competitive response (*log CR*) as a function of competitive effect (*log CE*).** Shown are pooled means across fertilizer levels. Error bars reflect one standard error of the mean. Regressions were conducted on species means and the p-values correspond to the regression model slope. For improved clarity, regressions for the second harvest were omitted.

We predicted that that initial biomass differences between Johnsongrass and target species, measured on single plants, would determine the magnitude of Johnsongrass’ competitive effect on target species. Competitive effects were indeed highly correlated with non-root biomass across harvest dates (Fig 3A). The correlation was strongest at the third harvest, at the end of the drought period, and somewhat less pronounced at the final harvest. The initial biomass of switchgrass and little bluestem was very similar and Johnsongrass competition reduced their non-root biomass in similar proportions relative to single plants. Big bluestem’s initial biomass was intermediate between those two species and Johnsongrass and the competitive effect of Johnsongrass was also intermediate.

Further, we predicted that competitive effect and response should be negatively correlated. At the first and third harvests, there was a significantly negative correlation between *log CE* and *log CR* (Fig 3B). This effect was generated by the difference between Johnsongrass and the native species, the former being weakly suppressed by competition with itself and the latter being strongly suppressed by Johnsongrass but in turn suppressing Johnsongrass weakly. Among the native species, *log CE* and *log CR* were not correlated, due to insufficient separation in either *logCE* or *logCR* at any given sampling date.

### Nitrogen relations

**Fig 4:**
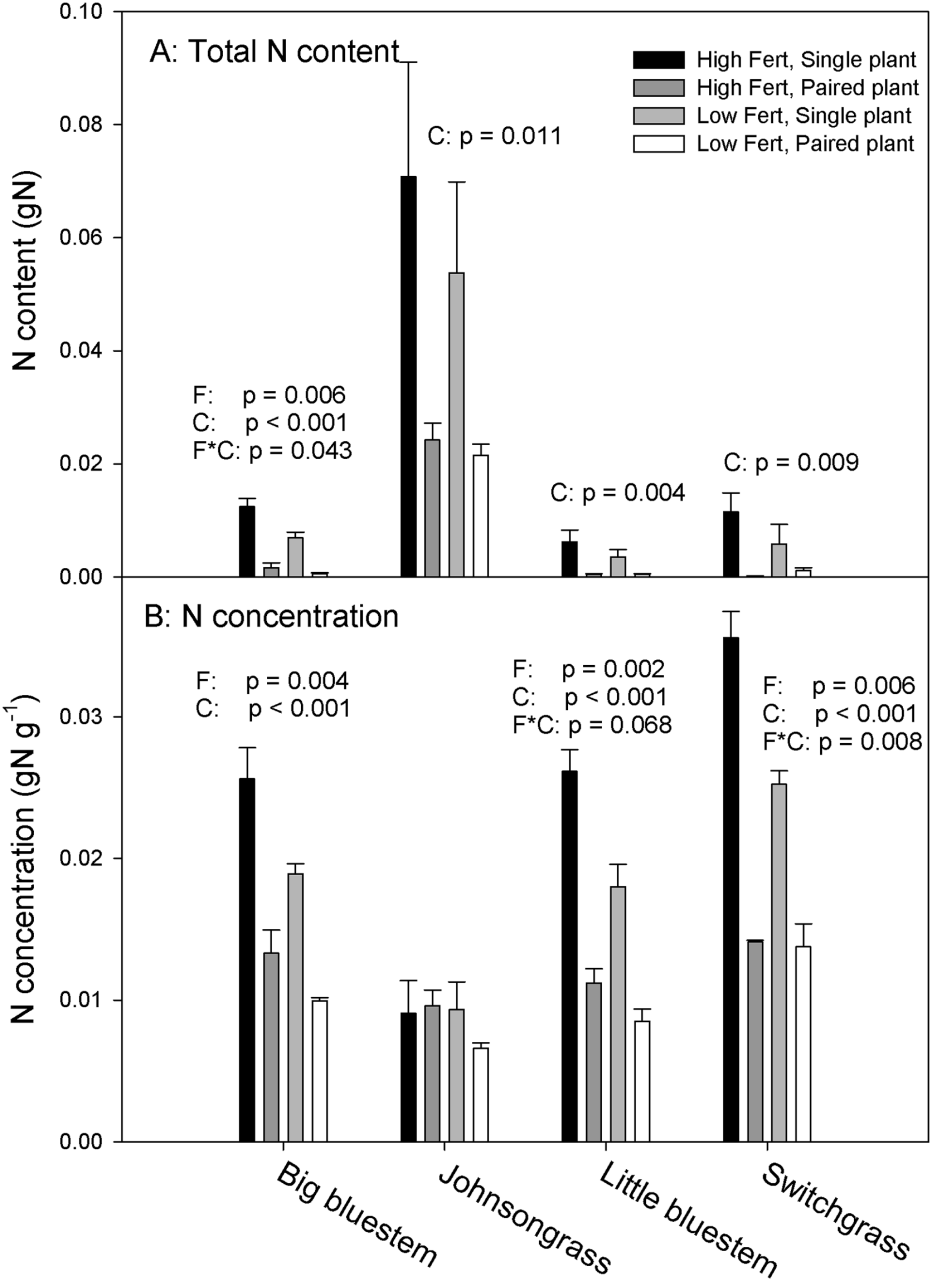
Total nitrogen content and nitrogen concentration in above-ground biomass at first harvest. Error bars reflect one standard error of the mean. The p-values of statistically significant or marginally significant effects are summarized by species in each panel. Letter symbols stand for the effects of fertilizer (F), competition (C) and their interaction (F*C).

The total nitrogen content of non-root biomass was significantly greater in Johnsongrass than in other species at first harvest (Fig 4A), following the trend in non-root biomass, but nitrogen concentrations were lower (Fig 4B). Fertilizer level neither affected the total amount of nitrogen in non-root biomass, nor its concentration. In the native species, both the lower fertilizer level and competition reduced nitrogen concentrations. In little bluestem and switchgrass the fertilizer effect was reduced in the paired treatment compared to single plants (fertilizer x competition interaction p = 0.068 and 0.008, respectively).

### Water relations

**Fig 5:**
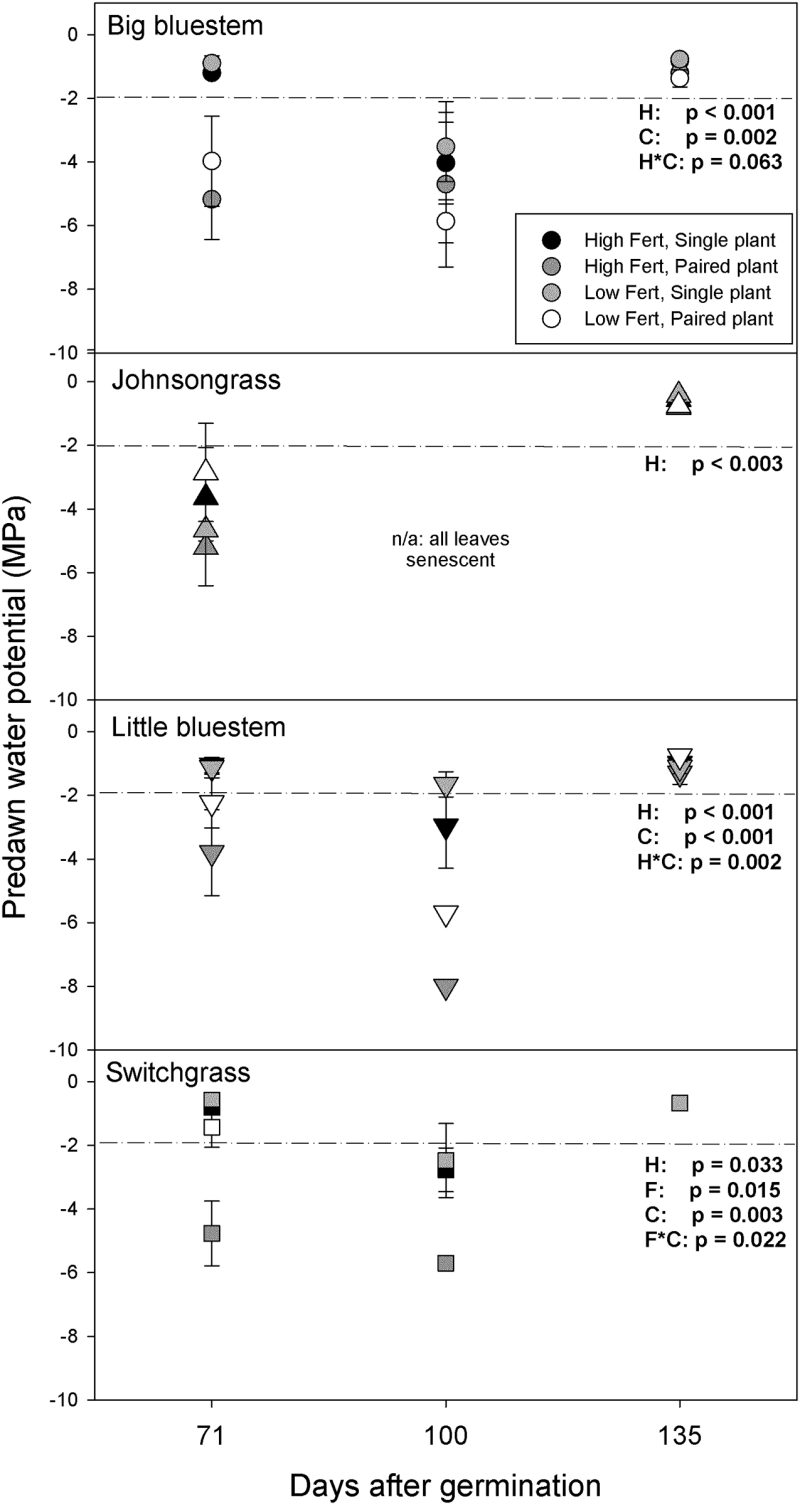
Predawn water potentials at approximately the mid-point (day 71) and the end (day 100) of the dry-down period and 35 days after resuming the watering regime (day 135). Error bars reflect one standard error of the mean. The p-values of statistically significant or marginally significant effects are summarized by species in each panel. Letter symbols stand for the effects of harvest date (H), fertilizer (F), competition (C) and their interactions (H*C, F*C). Where there is no error bar only one measurement could be obtained. Missing values indicate the absence of live leaf area. At water potential values below the dotted line at– 2MPa, gas exchange is severely restricted by stomatal conductance based on S1 Fig.

During the drought period between the first and third harvest, Ψ_pre_ values declined as expected and recovered when watering resumed (Fig 5). Half-way into the drought period, Johnsongrass grown single or paired had average Ψ_pre_ values well below the -2 MPa threshold, indicating a severe restriction of stomatal conductance (S1 Fig). At the end of the drought period, all Johnsongrass leaves had senesced, but green leaf area recovered after the drought. In consequence, only harvest date effects were significant in Johnsongrass, merely indicating the difference of mid-drought and post-drought leaf water potentials.

For the two bluestems, harvest date x competition effects were significant. Grown single, the bluestems had Ψ_pre_ values well above the -2 MPa threshold level and competition reduced Ψ_pre_ values to the threshold or below. In switchgrass this interaction was not significant, in part due to low sample size caused by the paucity of live leaves at the end of the drought. Unlike the two bluestems, switchgrass exhibited significant fertilizer effect and fertilizer*competition interaction, indicating that that the combination of the higher fertilizer level and competition caused the greatest water deficit.

## Discussion

### Competitive interactions

The competitive effect of Johnsongrass on native species was severe. Johnsongrass reduced the non-root biomass of native species to 5% or less of that of single plants, whereas Johnsongrass suffered an 11% loss of non-root biomass relative to single plants (Fig 2). Sharing warm-season C_4_ physiologies and seemingly similar morphological traits (S2 Fig), the overwhelming competitive advantage of Johnsongrass appeared to originated with its larger initial biomass, subsequently amplified by size-asymmetric resource competition [17]. We previously showed that Johnsongrass exceeded the biomass of native species by 4:1 as early as 17 days after germination [29]. In this experiment, 50 days after seeding, single Johnsongrass plants exceeded the biomass of single native plants by about 20:1 on average (Fig 1). At final harvest this ratio had declined to 1.4:1 for total biomass or 3:1 for non-root biomass. Thus, native species compensated for their slower growth in the first 50 days by growing faster later on (Fig 1B). In competition, however, no compensatory growth took place, rather, initial size disparities were amplified to 40:1 for non-root biomass at final harvest. Thus, size-asymmetric competition, disproportionally suppressing the growth of smaller individuals, was likely to be a major factor in producing the competitive suppression of native species.

As predicted, the competitive effect of Johnsongrass was directly propertional to the difference in initial biomass between Johnsongrass and target species (Fig 3A). Johnsongrass itself suffered the lowest reduction in biomass relative to single plants due to competition, about 50%, and the species with the smallest initial biomass the most. Although the magnitude of the competitive effects changed between harvests, species rankings (Johnsongrass < big bluestem < little bluestem/sitchgrass) were consistent over time.

We also hypothesized that competition between functionally similar species should approximate a “zero-sum game”, with constant yield (final biomass) irrespective of species composition. Therefore, we predicted a negative correlation between competitive effect and response. This prediction was supported, but mainly for the contrast between Johnsongrass and the native species (Fig 3B). The competitive response of Johnsongrass to the native species was simply too weak to produce a significant difference between native species. In direct support of the ‘constant yield’ assumption, the combined biomass of paired plants (shoots & roots) was not significantly affected by focal plant idenity (ANOVA p = 0.897).

Lastly, we predicted that a difference in soil fertility should not greatly change competitive interactions. The competitive effect was elevated at the higher fertilizer level (Fig 3), with the overall effect to reduce the final biomass of native species from 4.3% to 2.8% of single plant biomass, which may be considered a minor effect. We also noted a transient increase in native grass competition against Johnsongrass under low fertilizer at the third harvest, suggesting that competitiveness of natives may be higher under conditions more stressful than those present in this study. In general, however, these findings conform with other studies which found that variation in soil fertility was not a game changer for the competitive relationships between invasive and native species [34]. Rather, the increase in productivity may have intensified asymmetric competition and further amplified the size hierarchy between Johnsongrass and the native species [35].

### The origin of Johnsongrass’ early growth advantage

An early growth spurt in Johnsongrass set up the size advantage that assured its overwhelming competitive effect on the native species. The question is, how did Johnsongrass achieve this? We reported earlier that two-week old Johnsongrass seedlings had higher specific leaf area and photosynthetic nitrogen use efficiency than the native species, together with lower root:shoot ratios and leaf nitrogen density [29]. These traits are consistent with an early investment in accelerated leaf area growth, which tends to increase the growth potential in herbaceous plants [36]. Ontogenetic shifts continued with further growth, as investment shifted to lower SLA and higher root:shoot ratios, reflecting the need for structural biomass to support larger leaves and roots to provide needed water and nitrogen, at the cost of lower relative growth rates (Fig 3B). In addition, a larger plant may run faster out of critical resources, as was seen in this experiment during the mid-season drought, when water potentials were more rapidly declining for Johnsongrass. Thus, over a growing season, the benefit of accelerated early growth may be limited, *except* in the context of competition.

Accelerated growth relative to competitors deprives the competitors of resources, including water, nitrogen and light. Unfortunately, we did not measure light interception in this experiment, but under experimental conditions, shading would have been possible as paired plants grew only a few centimeters apart. However, we did document that Johnsongrass deprived native species of nitrogen and water, as native plants growing with Johnsongrass had lower nitrogen concentrations (Fig 4) and lower water potentials (Fig 5) than single plants.

Accelerated early growth may also compensate for the higher germination temperature requirement in Johnsongrass compared to the natives, giving Johnsongrass a mechanism by which to catch up to early-germinating natives. However, the implications of a potential trade-off between early germination at cool temperatures and faster seedling development at warm temperatures deserves further experimental investigation, and may be a mechanism by which inter-annual temperature variation predicts whether natives or Johnsongrass are favored in a given year.

### Drought response

It has been suggested that the downside of fast growth for plants in water-limited environments is the accelerated onset of water stress [21]. In this experiment, under the added constraint of a limited soil volume, Johnsongrass suffered more intense water stress than the native species and was more susceptible to leaf senescence (Fig 5). However, by the time that water was withheld on day 50, Johnsongrass had already invested in rhizome biomass, specifically, 6% of its total biomass was in rhizomes, equivalent to roughly 25% of the total biomass of the native species at that time. Most Johnsongrass plants added more biomass after the drought by resprouting from roots and rhizomes. Many of the native species, on the other hand, maintained green leaf area, which may have given native plants a small ‘window of opportunity’ during which their growth could outpace Johnsongrass, thereby stalling further competitive suppression or even slightly reversing it (Fig 3A). This suggests that variation in water availability and the timing of drought is likely to modify the competitive effect of Johnsongrass in native grasslands. Especially early drought might cut short the period over which Johnsongrass accrues its size advantage.

### Johnsongrass’ invasive traits

This study together with [29] documented that accelerated seedling growth, which places Johnsongrass at the top of a seedling size hierarchy among dominant C_4_ warm season grasses, could be an important aspect of Johnsongrass’ invasive success. Added to this, a rapid ontogenetic switch from accelerated leaf area growth to investment in rhizomes [37] and possible allelopathic effects [26] promote the persistence of established Johnsongrass patches. The competitive strength of recruits coupled with the persistence of adults is a powerful combination and one could argue that either a lack of competitiveness in the seedling stage or a lack of persistence in the mature stage would make Johnsongrass far less invasive: Smaller seedlings would reduce the ability to establish in grassland communities [38], while reduced persistence, at least theoretically, would make Johnsongrass more susceptible to population decline under unfavorable conditions.

This invasive strategy applies to ‘open sites’, i.e. areas that have undergone severe disturbance, such as by extreme fire or drought. Plant invasions often get started in disturbed areas, which has led to the hypothesis that invasive species require higher resource levels to be successful [39]. However, open sites also assure that seedlings compete against seedlings, which in the case of Johnsongrass, may be the key advantage. Future research should identify whether this advantage plays out in the field, and whether competitive dynamics can be altered by eliminating the size advantage by, for example, giving native grasses varying levels of a head start by staggering their germination and establishment.

Ontogenetic shifts between small juvenile stages and large adult stages are important niche components [40–42]. Species differences in growth patterns during early seedling development may be particularly pertinent in invasion ecology under the scenario we outlined here. Similar plant structure and a shared climatic niche would tend to select similar traits in the mature cormophyte, but developmental patterns in the embryo and seedling could be evolutionarily less constrained and give invasive species a distinct fitness advantage over members of the same niche.

For decades, researchers have asked what traits are associated with invasiveness. Comparing traits across a broad sweep of plant functional types, there may be no simple answer [6, 43]. But it helps to consider that in any community, competitive interactions are most intense between functionally similar species [8, 44], because similar species are most likely to compete head-on for the same resources including suitable, open sites. For winning the occupancy of open sites, the entry point for most invasions, there may be no better advantage than to produce larger seedlings, which suppress native species by resource preemption.

## Supporting information

S1 Fig Leaf conductance in relation to predawn water potentials measured the same day. Solid symbols for the higher Fertilizer level, open symbols for the lower level. The data were pooled from the July, August, September and October harvests.
(TIF)

S2 Fig Trait values at first harvest for plants grown alone. A: Median root diameter (MRD), B: Specific Leaf Area (SLA), C: leaf photosynthetic rate (A), D: Leaf nitrogen density (LND) and E: Log root:shoot (Log R:S). S stands for species effects and F for Fertilizer effects on trait values. Only significant (p ≤ 0.05) or marginally significant (0.05 > p > 0.1) p-values are shown. Solid symbols for the higher fertilizer, open symbols for the lower level. Error bars reflect one standard error of the mean.
(TIF)

S1 File Biomass data. The first 8 columns contain treatment information. ‘Harvest Date’ indicates the month in which the harvest was conducted, ‘ID’ is a unique plant identifier, ‘Species’ refers to the species for which biomass values are shown. ‘Species B’ refers to the species combination, ‘Big’, ‘John’, ‘Little’ and ‘Switch’ refer to big bluestem, Johnsongrass, little bluestem and switchgrass, respectively, grown alone. Combinations of these species identifiers refer to the respective species grown together, where the first species name identifies the species for which the data are shown. For example, ‘JohnBig’ and ‘BigJohn’ refer to the same treatment in which a big bluestem target plant is grown with a Johnsongrass neighbor, but the first ‘Species B’ value indicates that Johnsongrass data are shown, the second ‘Species B’ value indicates that big bluestem data are shown. ‘Nitro’ refers to the fertilizer treatment, ‘Focal Spc.’ indicates whether the data are shown for the focal plant (‘Yes’) or the neighbor (‘No’), ‘Combo Pot’ indicates whether the treatment involves one plant grown alone (‘No’) or two plants grown together (‘Yes’). All biomass values are shown in grams. ‘Root Wt.’ is the root weight for plants grown alone, ‘Combo Root Wt.’ is the combined root weight for two plants grown together, ‘Rhizone Wt.’ is the weight of rhizome biomass, ‘Leaf (s) Wt.’ is the weight of senesced leaves, ‘Leaf (g) Wt.’ is the weight of green leaves, ‘Reprod. Wt.’ is the weight of flowers and seeds, ‘Old Stem Wt.’, ‘Old Leaf Wt.’ and ‘Old Reprod. Wt.’ are the weights of the respective biomass components that had died off in the course of the drought and ‘Total Old’ is the sum of these components. ‘Total Above Ground’ is the sum of live and old leaf, stem, rhizome and reproductive biomass, ‘Total Wt’ is the sum of all biomass components.
(XLSX)

S2 File Gas exchange and plant water potential data. The first 7 columns contain treatment information. ‘Harvest Date’ indicates the month in which the harvest was conducted, ‘ID’ is a unique plant identifier, ‘Species’ refers to the species for which data are shown. ‘Big’, ‘John’, ‘Little’ and ‘Switch’ refer to big bluestem, Johnsongrass, little bluestem and switchgrass, respectively. ‘Nitro’ refers to the fertilizer treatment, ‘Focal Spc.’ Indicates whether the data are shown for the focal plant (‘Yes’) or the neighbor (‘No’), ‘Combo Pot’ indicates whether the treatment involves one plant grown alone (‘No’) or two plants grown together (‘Yes’). ‘Photo’ is the leaf photosynthetic rate in micro-mol CO_2_ m^-2^ s^-1^, ‘Cond’ is the leaf conductivity in mol H_2_O m^-2^ s^-1^, ‘Ci’ is the intercellular CO_2_ concentration in ppm, ‘Water Potential’ is the predawn water potential measured on leaf blades in MPa.
(XLSX)

S3 File Nitrogen content at first harvest in July. The first 6 columns contain treatment information. ‘ID’ is a unique plant identifier, ‘Species’ refers to the species for which data are shown. ‘Big’, ‘John’, ‘Little’ and ‘Switch’ refer to big bluestem, Johnsongrass, little bluestem and switchgrass, respectively. ‘Species B’ refers to the species combination, ‘Big’, ‘John’, ‘Little’ and ‘Switch’ refer to big bluestem, Johnsongrass, little bluestem and switchgrass, respectively, grown alone. Combinations of these species identifiers refer to the respective species grown together, where the first species name identifies the species for which the data are shown. For example, ‘JohnBig’ and ‘BigJohn’ refer to the same treatment in which a big bluestem target plant is grown with a Johnsongrass neighbor, but the first ‘Species B’ value indicates that Johnsongrass data are shown, the second ‘Species B’ value indicates that big bluestem data are shown. ‘Nitro’ refers to the fertilizer treatment, ‘Focal Spc.’ indicates whether the data are shown for the focal plant (‘Yes’) or the neighbor (‘No’), ‘Combo Pot’ indicates whether the treatment involves one plant grown alone (‘No’) or two plants grown together (‘Yes’). ‘SLA’ is the specific leaf area in g cm^-2^, ‘Leaf area’ is the total leaf area per plant in cm^2^, ‘N/area’ is the total leaf nitrogen density in g cm^-2^, the next three columns are the root weight in g, their % N content by mass and their total N content in g. The next three columns are the combined root weights of two plants in the competition treatment in g, their % N content by mass and their total N in g. The next three columns are the rhizome weight in g, their % N content by mass and their total N content in g. The next three columns are the stem weight in g, their % N content by mass and their total N content in g. The next three columns are the weight of senesced leaves in g, their % N content by mass and their total N content in g. The next three columns are the weights of green leaves in g, their % N content by mass and their total N content in g. The last three columns are the weights of flowers and seeds in g, their % N content by mass and their total N content in g.
(XLSX)
